# Pallister-Killian syndrome: clinical, cytogenetic and molecular findings in 15 cases

**DOI:** 10.1186/s13039-018-0395-z

**Published:** 2018-08-17

**Authors:** Birsen Karaman, Hülya Kayserili, Asadollah Ghanbari, Zehra Oya Uyguner, Güven Toksoy, Umut Altunoglu, Seher Basaran

**Affiliations:** 1 Department of Medical Genetics, Istanbul Medical Faculty, Istanbul University, Millet cad.34039 Capa, İstanbul, Turkey; 2 Medical Genetics Department & Genetic Diagnosis Center, Koç University School of Medicine, 34010 İstanbul, Turkey

**Keywords:** OMIM 601803, Pallister-Killian syndrome, Somatic mosaicism, Mosaic tetrasomy 12p, Isochromosome 12p, Parental origin, Small supernumerary marker chromosome

## Abstract

**Background:**

Pallister Killian syndrome (PKS, OMIM 601803) is a rare genetic disorder with a distinct phenotype caused by tissue- limited mosaicism tetrasomy of the short arm of chromosome 12, which usually cytogenetically presents as an extra isochromosome 12p.

Wide phenotypic variability in PKS has been reported, ranging from pre-to perinatal death due to multiple congenital anomalies, especially diaphragmatic hernia, and classic phenotypes including seizures, severe developmental delay, macrosomia at birth, deafness, and distinct dysmorphic features, such as coarse face, temporal alopecia, a small nose with anteverted nostrils, long philtrum, and hypo−/hyper- pigmented streaks on the skin.

**Results:**

Karyotypes obtained from cultured peripheral lymphocytes of 13 cases, who were diagnosed as PKS, were normal, while karyotypes obtained from cultured skin samples and buccal mucosa revealed the supernumerary mosaic i(12p). Mosaic karyotype was found in both fibroblast and buccal mucosa in 14 of 15 patients in our series, whereas in one stillbirth, following the clinical diagnosis of PKS, skin and buccal smear samples were taken, and all karyotypes from cultured fibroblasts revealed a supernumerary i(12p), while I-FISH study showed 60% mosaicism in mucosal cells.

**Conclusions:**

We here share the clinical, cytogenetic and molecular cytogenetic findings of 15 cases with PKS phenotype and the parental origin of seven i(12p) identified by molecular analyses. To our knowledge, this is the largest series of PKS patients with parental origin study from a single center. We believe that our study makes a significant contribution to the literature because we specifically found no differences in the phenotypes of cases with either a maternal or paternal origin of the extra element and differential imprinting appeared not to be a factor.

## Background

Pallister-Killian syndrome (PKS) was first described an adult phenotype by Pallister in 1977 and independently a childhood phenotype by Killian and Teschler-Nicola in 1981 [1, 2]. In both instances, cytogenetic examinations of blood cells did not reveal any abnormality. Later observations by Schinzel in 1991 confirmed a distinct phenotype. In 1987, PKS was shown to be caused by somatic mosaicism of an extra chromosome, which was not primarily not detected since mosaicism was limited to fibroblasts and not present in lymphocytes [3, 4]. Using banded karyotypes, and after several mis-interpretations, the extra element was identified as an isochromosome 12p.

The clinical phenotype of PKS is quite homogeneous for the dysmorphic pattern, but highly variable for developmental delay, congenital malformations, and survival. There is a wide range of congenital malformations that occur rarely including anal atresia/stenosis and atlanto-occipital fusion. Areas of hyper- or hypo-pigmentation are often found and reflect the mosaic pattern of the skin, with different proportions of hyperdiploid cells [5].

The developmental delay in patients with PKS is quite variable, and although most reported patients with PKS have severe to profound mental retardation, milder phenotypes have also been reported [6–13]. A large number of patients with i(12p) were reported in the literature, and also their clinical and cytogenetic information routinely posted in a database (http://ssmc-tl.com/sSMC.html http://ssmc-tl.com/chromosome-12.html http://ssmc-tl.com/chromosome-12.html#iso) [14].

Here, we report the cytogenetic, and molecular cytogenetic findings in 15 cases with PKS phenotype and parental origin analysis results of 7 cases with i(12p) chromosome. To our knowledge, this series is the first series from Turkey and the largest series of PKS patients with parental origin study from a single center.

## Methods

**Fig. 1:**
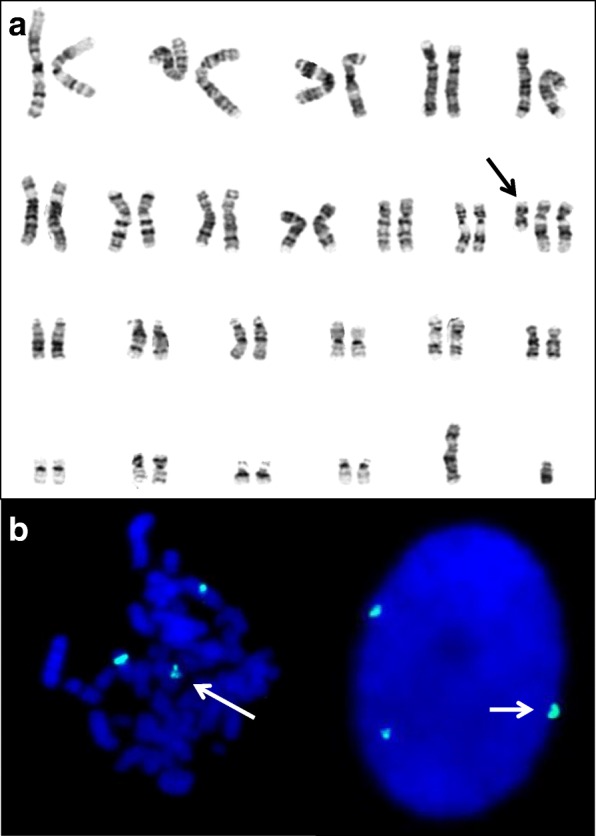
**a** G-banding karyotype of the fibroblasts. The arrow shows the isochromosome 12p. **b** FISH analysis using 12 centromere specific D12Z3 probe (Aquarius®, Cytocell Cambridge, UK) in the fibroblasts. The white arrow shows a supernumerary chromosome in the metaphase and interphase

All cases were evaluated at Medical Genetics Department outpatients’ clinics, Istanbul Medical Faculty, Istanbul University. Cytogenetic and molecular cytogenetic analyses were performed in the laboratory of the department. Before the implementation of the FISH technique, in presence of the clinical features of PKS, cytogenetic studies were performed from cultured fibroblasts from skin biopsies in parallel to the peripheral lymphocyte cultures. Following the introduction of the interphase FISH (I-FISH) study using chromosome 12 centromeric probe (D12Z3, Aquarius®, Cytocell Cambridge, UK), I-FISH test on mucosal cells from buccal smear were applied- in parallel to the lymphocyte cultures (Fig. 1).

De novo supernumerary i(12p) was further confirmed by FISH using chromosome 12 specific wcp, centromeric and subtelomeric probes.

To determine the parental origin and formation mechanism, 7 cases with i(12p) were investigated with gel-electrophoresis technique using 15 microsatellite markers on 12p (D12S352, D12S99, D12S77, D12S269, D12S320, D12S364, D12S1303, D12S373, D12S301, D12S1669, D12S1650, D12S799, D12S823, D12S1053, D12S345,) and 2 markers on 12q (D12S72, D12S43).

## Results

### Clinical findings

**Fig. 2:**
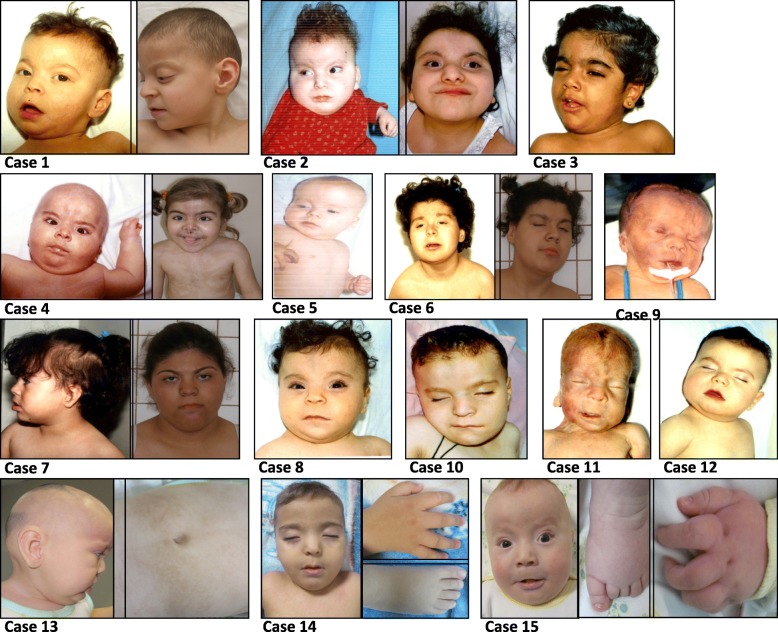
Facial appearance of the patients. Alopecia/Sparse temporal hair, hypo- or hyper pigmented areas and other stigmata such as epicanthic folds and slightly low-set ears, prominent forehead, hypertelorism, up- slanting palpebral fissures, anteverted nostrils, long philtrum, everted lower lip, short neck and short hands and toes

Fifteen cases were cytogenetically evaluated due to their phenotypic features (Fig. 2).

The mean age at the time of diagnosis was about 2.5 years of age (ranging between one day-7 years 10/12).

Karyotypes of fibroblast cultures in 15 cases revealed the supernumerary mosaic i(12p), while cultured peripheral blood lymphocytes of those cases were normal. The 12 p tetrasomic metaphases were observed 10% to 100% of cells analyzed in fibroblasts. Although the level of +i(12p) in cultured fibroblasts was low (11%), I-FISH indicated higher level of i(12p) (40%) in the mucosal cells in one case. In one stillborn, following the clinical diagnosis of PKS, skin and buccal smear samples were taken, and a supernumerary i(12p) was present in all metaphases of cultured fibroblasts, while I-FISH study showed mosaicism of 60% in mucosal cells (Table 1).

**Table 1:** Karyotypes and FISH findings in different tissues

Case no	Karyotypes on cultured lymphocytes	Karyotype on fibroblast	%	I-FISH results on buccal swap sample	%	Parental origin
1	46,XY	mos 46,XY/47,XY,+i(12)(p10)[33/17]	34	–		P
2	46,XX	mos 46,XX/47,XX,+i(12)(p10)[18/2]	10	–		M
3	46,XX	mos 46,XX/47,XX,+i(12)(p10)[100/11]	9.9	.ish (D12Z2x2/D12Z2x3)[50/13]	20.6	
4	46,XX	mos 46,XX/47,XX,+i(12)(p10)[28/12]	30	–		
5	46,XY	mos 46,XY/47,XY,+i(12)(p10)[65/35]	35	–		M
6	46,XX	mos 46,XX/47,XX,+i(12)(p10)[24/9]	27.3	–		M
7	46,XX	mos 46,XX/47,XX,+i(12)(p10)[15/5]	25	–		M
8	46,XX	mos 46,XX/47,XX,+i(12)(p10)[31/4]	11.4	.ish (D12Z2x2/D12Z2x3)[64/41]	39	
9	46,XY	mos 46,XY/47,XY,+i(12)(p10)[21/9]	30	.ish (D12Z2x2/D12Z2x3)[5/13]	72	
10	46,XY	mos 46,XY/47,XY,+i(12)(p10)[20/20]	50	.ish (D12Z2x2/D12Z2x3)[25/30]	54.5	
11	–	47,XY,+i(12)(p10)	100	.ish (D12Z2x2/D12Z2x3)[40/60]	60	M
12	46,XY	mos 46,XY/47,XY,+i(12)(p10)[25/5]	16.6	.ish (D12Z2x2/D12Z2x3)[7/25]	78.1	M
13	47,XY,+mar.ish(14/22)(q10;q10)mat	48,XY,+mar.ish(14/22)(q10;q10) mat, +i(12(p10)	100	–		
14	–	mos 46,XY/47,XY,+i(12)(p10)[45/40]	47	.ish (D12Z2x2/D12Z2x3)[71/68] (F) .ish (D12Z2x2/D12Z2x3)[50/53] (BS)	49 51.4	
15	46,XY	–		mos.ish (D12Z2x2/D12Z2x3)[180/20]	10	

*F* Fibroblast, *BS* Buccal swap, *M* Maternal, *P* Paternal

The clinical findings of the cases with tetrasomy 12p are summarized in Table 2. Almost all cases with tetrasomy 12p had coarse facial features, bitemporal alopecia, anteverted nostrils and everted lower lip, hypertelorism, epicanthus, long philtrum and hypo/hyperpigmented skin changes. Intellectual disability was observed in all patients over 8 months of age except one case with near normal intelligence at adult age (case 7), and three newborns.

**Table 2:** Clinical finding of Pallister Killian patients

Case No	1	2	3	4	5	6	7	8	9	10	11	12	13	14	15
Sex	m	f	f	f	m	f	f	f	m	m	m	m	m	m	m
Age at diagnosis (days/months (m)/year)	7 ^7/12^	10 ^8/12^	6 ^8/12^	12 ^10/12^	9 m	7 ^1/12^	2 ^11/12^	8 m	1 day	5 ^10/12^	22 days	11 m	1 day	1	1^1/12^
Gestation age at bırth	‘term’	38	37	40	39	‘term’	‘term’	36	33	41	37	‘term’	40	32	38
Maternal/paternal age at conception	18/25	32/33	22/22	23/28	28/29	38/38	19/25	35/42	31/31	21/23	33/31	28/29	39/33	32/32	33/41
Weight/length at bırth	2100/?	3500/47	3500/50	2600/45	3450/54	4250/?	3250/?	2250/45	2500/44	4750/?	2950/49	3800/	3200/53	2800/?	3830/53
Intellectual disability	+	+	+	+	+	+	-(near norma)	+	?	+	?	+	?	+	+
Seizures	–	–	+	+	–	–	–	–	–	–	–	–	–	+	+
Hypotonia	+	+	+	–	+	+	–	+	–	–	–	–	+	+	+
Prominent forehead	+	+	–	+	+	+	+	+	–	–	–	+	+	+	+
Bitemporal alopecia/sparse temporal hair	+	+	+	+	+	+	+	+	+	+	+	+	+	+	+
Coarse facial features	+	+	+	+	+	+	+	+	+	+	+	+	+	+	+
Flat occiput	+	–	–	–	–	–	–	–	–	–	–	–	–	+	–
Hypertelorism	+	+	borderline	+	+	+	–	+	–	–	+	–	+	+	+
Epicanthus	–	–	–	–	–	–	–	–	–	–	–	–	+	+	–
Dysmorphic ears	+	+	–	–	+	+	–	+	+	+	+	+	+	+	+
Short nose	–	–	+	–	+	–	+	+	+	+	+	+	+	+	+
Anteverted nostrils	+	–	+	+	+	+	+	+	+	+	+	+	+	+	+
Long philtrum	+	–	–	+	+	–	+	+	+	+	+	+	+	+	+
Macroglossia	–	–	–	–	–	–	–	+	–	–	–	–	–	+	
Micrognathia	–	–	–	–	+	–	–	+	–	–	–	+	+	+	–
Everted lower lip	+	+	+	+	+	+	+	+	+	+	+	+	+	+	+
Short neck	–	+	+	+	+	–	+	–	+	+	–	–	+	+	+
Accessory nipples	–	–	–	+	–	–	–	+	–	–	–	–	–	–	–
Hypo/hyperpigmented areas of skın	+/+	+/+	+/+	+/+	+/+	+/+	+/+	+/+	−/−	+/+	−/−	+/+	+/+	+/+	−/−
Short hands and feet	+	+	–	+	+	–	+	+	–	+	–	+	–	+	+
CHD	–	–	–	PDA,PFO,VSD	–	–	–	–	PDA	–	PFO, PDA	–		ASD	–

*Abbr*: *CHD* congenital heart defect, *PDA* patent ductus arteriosus, *PF O* patent foramen ovale, *VSD* ventricular septal defect, *ASD* atrial septal defect

Parental origin was determined by STR analysis as maternal in 6 cases (85.7%), paternal in one case (14.22%).

## Discussion

Classical PKS is a rare condition caused by mosaic tetrasomy 12p presenting with an additional isochromosome of 12p, which is usually not present in blood lymphocyte cultures, but can be shown in skin fibroblasts [3, 4] and other tissues such as buccal smears, chorionic villi and amniocytes. The clinical phenotype is characterized by a pattern of dysmorphic features, hypo- or hyperpigmentation areas of the skin, progressive coarsening of the face with advancing age and moderate to severe- profound intellectual disability accompanied with different types of seizures. Congenital malformations have been found in a minority of live births.

The dysmorphic pattern is age-dependent and includes brachycephaly, temporal balding during the first years of life, a short nose with flat bridge and anteverted nostrils giving the false impression of hypertelorism, microstomia, progressive macroglossia, prominent everted lower lip, small mandible and short neck. Supernumerary nipples are frequent as are hypoplastic male external genitalia. Hands and fingers, feet and toes are proportionately small. Pigmentation anomalies of the skin are due to mosaicism with different expression of pigmentation genes with predominance of one or the other cell line. This is well known for mosaicism in general [14–23].

Although there are limited number of reports on the mosaicism of the isochromosome in peripheral blood cells in PKS patients by I-FISH, microarray studies, and karyotype analysis [24–27]. Studies have shown that phytohaemagglutinin used in lymphocyte cultures promotes the growth of the normal cells, which leads to under- representation or disappearing of the abnormal cells [28–31]. Furthermore tissues with a shorter turnover period seem to lose the cells with additional chromosomes, either due to longer duration of cell division, the instability of (submicroscopically) dicentric chromosomes or the abnormal cell line going through apoptosis or necrosis at a higher rate than the normal cells [32]. At present I-FISH using chromosome 12 centromeric probes on buccal mucosa cells is a very useful, non-invasive, reliable, rapid and cost-effective test for the definite diagnosis [33, 34]. It is suggested that all live born tetrasomy 12 cases are mosaic, even it could not be shown in one tissue. Therefore, it is helpful to investigate different tissues by different techniques to enlighten the mosaicism like in our case 11 (Table 2). However, in all similar cases from the literature only one or at most two different tissues had been analysed and thus mosaicism in non-examined tissues or even in a second skin biopsy from another area of the body was not excluded. In a few cases with clinical diagnosis of PKS normal karyotype both in blood and fibroblast cultures derived from one or two skin biopsies mosaicism could not be excluded.

### Early cytogenetic misinterpretations

Prior to and during the early banding era of cytogenetics, several patients were published as tetrasomy 21 (an aberration which seems incompatible with life) [35–39] and the extra chromosome was also classified as chromosome 12 with deletion of about ¾ of the long arm [40].

### Mechanism (s) of formation of the additional i(12p)

Molecular marker analysis favored the following steps: Initial maternal meiosis II nondisjunction (evidenced by centromere-near homozygosity of the maternal markers) leading to trisomy 12. Trisomy 12 zygotes will spontaneously abort; the few exceptions are those conceptuses in which a secondary structural aberration, isochromosome formation, results in loss of the acentric long arms and subsequent formatıon of an +i(12p) [41–45]. Ravel et al. published a mosaic case with trisomy 12 and + i(12p) diagnosed prenatally and proposed a formation mechanism for +i12p [46]. Pauli et al. discussed the mechanisms of formation based on the data of their monozygotic twins and suggested that prezygotic or postzygotic error (both before the twinning event) in the inner cell mass may have happened. [47].

The increased mean maternal age at conception suggests this mechanism [43]. In a few cases paternal origin of the i(12p) could be demonstrated [25, 42, 48], obviously caused by a different cascade of events. To the best of our knowledge, there seems to be no difference in phenotype between the cases of maternal versus paternal origin of the extra element and thus no effect of differential imprinting [6, 10].

### Karyotype-phenotype correlation

It has been suggested that no correlation could be established between the proportion of tetrasomic cells and the severity of clinical presentation [5]. Our observation supports this suggestion, because of the technical limitations, the severity of the phenotype was independent from the degree of mosaicism in karyotyping. However, fibroblast cultures were analyzed from only one biopsy in all cases. As mentioned above, the incidence of hyperdiploid cells may grossly vary among fibroblast cultures, derived from different areas of pigmentation state even in the same individual [6, 10, 49]. Moreover, tissues whose mosaic constitution is the most important for the degree of developmental delay, i.e. CNS cells, cannot be investigated.

To date a few cases have been reported with only mild psychomotor delay and minor facial anomalies [49]. In our series, only one patient (case 7) presented with minor facial manifestations and near normal intelligence and after 20 years of follow up she has married and after an uneventful pregnancy had a normal baby.

The mechanism of the formation of isochromosomes is still not fully clear. Various mechanisms during prezygotic mitosis or meiosis or even postzygotic mitosis have been proposed. Hunter et al. proposed five different mechanisms for the formation of i(12p) and the issue was summarized by Struthers et al. [45, 50].

Our study was in agreement with the literature data on predominance of maternal inheritance in + i(12p) cases [42, 45, 46, 48, 51, 52].

It has been reported that the risk for +i(12p) increases with advanced maternal age [53–55]. The mean maternal age was 28.6 years (range: 18–39) in our cohort, however maternal age was over 33 for all the cases with maternal origin. Our findings are in agreement with the hypothesis that the increase in risk of having +i(12p) conceptus correlates with advanced maternal age.

## Conclusion

Phenotypic and cytogenetic variability of PKS may be challenging for the differential diagnosis. Karyotyping on lymphocytes usually do not reveal the supernumerary i(12p). Therefore, we suggest that in cases with suspicion of clinical diagnosis of PKS, when pigmentation anomalies of the skin and temporal balding is noted as accompanying features, I-FISH on buccal mucosa cells and karyotyping from peripheral lymphocytes should be performed as a first tier test.

## Abbreviations

CNS: Central Nervous System
FISH: Florescence In Situ Hybridization
I-FISH: Interphase FISH
PKS: Pallister-Killian syndrome
STR: Short Tandem Repeats

## References

[1] Pallister PD (1977). The pallister mosaic syndrome. Birth Defects Orig Artic Ser.

[2] Teschler-Nicola M, Killian W (1981). Case report 72: mental retardation, unusual facial appearance, abnormal hair. Synd Ident.

[3] Peltomäki P (1987). Pallister-Killian syndrome: cytogenetic and molecular studies. Clin Genet.

[4] Warburton D, Anyane-Yeboa K, Francke U (1987). Mosaic tetrasomy 12p: four new cases, and confirmation of the chromosomal origin of the supernumerary chromosome in one of the original Pallister-mosaic syndrome cases. Am J Med Genet.

[5] Schinzel A (1991). Tetrasomy 12p (Pallister-Killian syndrome). J Med Genet.

[6] Bielanska MM, Khalifa MM, Duncan AM (1996). Pallister-Killian syndrome: a mild case diagnosed by fluorescence in situ hybridization. Review of the literature and expansion of the phenotype. Am J Med Genet.

[7] Blyth M (2015). Pallister-Killian syndrome: a study of 22 British patients. J Med Genet.

[8] Filloux FM (2012). Occurrence and clinical features of epileptic and non-epileptic paroxysmal events in five children with Pallister-Killian syndrome. Eur J Med Genet.

[9] Kostanecka A (2012). Developmental and behavioral characteristics of individuals with Pallister-Killian syndrome. Am J Med Genet A.

[10] Schaefer GB (1997). Clinical variability of tetrasomy 12p. Clin Genet.

[11] Vogel I (2009). Pallister-Killian syndrome in a girl with mild developmental delay and mosaicism for hexasomy 12p. Am J Med Genet A.

[12] Izumi K, Krantz ID (2014). Pallister-Killian syndrome. Am J Med Genet C Semin Med Genet.

[13] Wilkens A (2012). Novel clinical manifestations in Pallister-Killian syndrome: comprehensive evaluation of 59 affected individuals and review of previously reported cases. Am J Med Genet A.

[14] Liehr, T., Small supernumerary marker chromosomes. 2018*.* http://ssmc-tl.com/sSMC.html, http://ssmc-tl.com/chromosome-12.html, http://ssmc-tl.com/chromosome-12.html#iso [accessed 08/07/2018]. 2018.

[15] Chitayat D, Friedman JM, Johnston MM (1990). Hypomelanosis of Ito--a nonspecific marker of somatic mosaicism: report of case with trisomy 18 mosaicism. Am J Med Genet.

[16] Donnai D, Read AP (1992). Hypomelanosis of Ito. Lancet.

[17] Donnai D (1988). Hypomelanosis of Ito: a manifestation of mosaicism or chimerism. J Med Genet.

[18] Happle R (1978). Genetic interpretation of linear skin abnormalities. Hautarzt.

[19] Harrod MJ, Howard-Peebles PN, Friedman JM. Tetrasomy 12p: differential degree of mosaicism in fibroblasts from normal and abnormally pigmented skin. Proc Greenwood Genet Center. 1986;6:107.

[20] Liehr T (2013). Clinical impact of somatic mosaicism in cases with small supernumerary marker chromosomes. Cytogenet Genome Res.

[21] Read AP, Donnai D (1990). Association of pigmentary anomalies with chromosomal and genetic mosaicism and chimerism. Am J Hum Genet.

[22] Thomas IT (1989). Association of pigmentary anomalies with chromosomal and genetic mosaicism and chimerism. Am J Hum Genet.

[23] Ward BE, Hayden MW, Robinson A (1988). Isochromosome 12p mosaicism (Pallister-Killian syndrome): newborn diagnosis by direct bone marrow analysis. Am J Med Genet.

[24] Cobben JM, Engelen M, Polstra A (2013). Array CGH on unstimulated blood does not detect all cases of Pallister-Killian syndrome: buccal smear analysis should remain the diagnostic procedure of first choice. Am J Med Genet A.

[25] Conlin LK (2012). Utility of SNP arrays in detecting, quantifying, and determining meiotic origin of tetrasomy 12p in blood from individuals with Pallister-Killian syndrome. Am J Med Genet A.

[26] Harrison V (2011). Diagnosis of Pallister-Killian syndrome by array comparative genome hybridization from a spleen sample. Clin Dysmorphol.

[27] Lloveras E (2013). Supernumerary ring chromosome: an etiology for Pallister-Killian syndrome?. Fetal Diagn Ther.

[28] Kayser M (2000). Blaschkolinear skin pigmentary variation due to trisomy 7 mosaicism. Am J Med Genet.

[29] Kulharya AS, Lovell CM, Flannery DB (2002). Unusual mosaic karyotype resulting from adjacent 1 segregation of t(11;22): importance of performing skin fibroblast karyotype in patients with unexplained multiple congenital anomalies. Am J Med Genet.

[30] Lessick ML, Szego K, Wong PW (1988). Trisomy 22 mosaicism with normal blood chromosomes. Case report with literature review. Clin Pediatr (Phila).

[31] Pagon RA (1979). Abnormal skin fibroblast cytogenetics in four dysmorphic patients with normal lymphocyte chromosomes. Am J Hum Genet.

[32] Tang W, Wenger SL (2005). Cell death as a possible mechanism for tissue limited mosaicism in Pallister-Killian syndrome. J Assoc Genet Technol.

[33] Manasse BF (2000). The Pallister-Killian syndrome is reliably diagnosed by FISH on buccal mucosa. Clin Dysmorphol.

[34] Ohashi H, Ishikiriyama S, Fukushima Y (1993). New diagnostic method for Pallister-Killian syndrome: detection of i(12p) in interphase nuclei of buccal mucosa by fluorescence in situ hybridization. Am J Med Genet.

[35] Chiesa J (1998). Pallister-Killian syndrome [i(12p)]: first pre-natal diagnosis using cordocentesis in the second trimester confirmed by in situ hybridization. Clin Genet.

[36] Fryns JP (1982). Mosaic tetrasomy 21 in severe mental handicap. Eur J Pediatr.

[37] Gilgenkrantz S (1985). Mosaic tetrasomy 12p. Clin Genet.

[38] Kunz J (2009). Tetrasomy 12p (Pallister-Killian syndrome): difficulties in prenatal diagnosis. Arch Gynecol Obstet.

[39] Park IY (2009). Prenatal diagnosis of Pallister-Killian syndrome associated with pulmonary stenosis and right ventricular dilatation. Korean J Lab Med.

[40] Karki CB, Walters RM (1990). Trisomy 12p mosaicism syndrome. J Ment Defic Res.

[41] Bugge M (1996). Tetrasomy 18p de novo: parental origin and different mechanisms of formation. Eur J Hum Genet.

[42] Cormier-Daire V (1997). Prezygotic origin of the isochromosome 12p in Pallister-Killian syndrome. Am J Med Genet.

[43] Kotzot D (1996). Isochromosome 18p results from maternal meiosis II nondisjunction. Eur J Hum Genet.

[44] Shen JD (2010). Pallister-Killian syndrome: meiosis II non-disjunction may be the first step in the formation of isochromosome 12p. Chin Med J.

[45] Struthers JL, Cuthbert CD, Khalifa MM (1999). Parental origin of the isochromosome 12p in Pallister-Killian syndrome: molecular analysis of one patient and review of the reported cases. Am J Med Genet.

[46] de Ravel TJ (2004). Post-zygotic origin of isochromosome 12p. Prenat Diagn.

[47] Pauli S (2012). Discordant phenotype in monozygotic twins with mosaic trisomy 12p in lymphocytes. Eur J Med Genet.

[48] Turleau C (1996). Parental origin and mechanisms of formation of three cases of 12p tetrasomy. Clin Genet.

[49] Genevieve D (2003). Mild phenotype in a 15-year-old boy with Pallister-Killian syndrome. Am J Med Genet A.

[50] Hunter AG, Clifford B, Cox DM (1985). The characteristic physiognomy and tissue specific karyotype distribution in the Pallister-Killian syndrome. Clin Genet.

[51] Los FJ (1995). Prenatal diagnosis of mosaic tetrasomy 12p/trisomy 12p by fluorescent in situ hybridization in amniotic fluid cells: a case report of Pallister-Killian syndrome. Prenat Diagn.

[52] Schubert R (1997). Report of two new cases of Pallister-Killian syndrome confirmed by FISH: tissue-specific mosaicism and loss of i(12p) by in vitro selection. Am J Med Genet.

[53] Rivera H, Rivas F, Cantu JM (1986). On the origin of extra isochromosomes. Clin Genet.

[54] Van Dyke DL, Babu VR, Weiss L (1987). Parental age, and how extra isochromosomes (secondary trisomy) arise. Clin Genet.

[55] Wenger SL, Boone LY, Steele MW (1990). Mosaicism in Pallister i(12p) syndrome. Am J Med Genet.

